# Sebaceous Filaments

**DOI:** 10.5826/dpc.1101a148

**Published:** 2021-01-29

**Authors:** Yuvrah Singh, Shekhar Neema, Amit Bahuguna, Disha Dabbas

**Affiliations:** 1 Department of Dermatology, Armed Forces Medical College, Pune, India

**Keywords:** sebaceous filaments, Demodex, mites, dermoscopy

## Case Presentation

**Figure 1:**
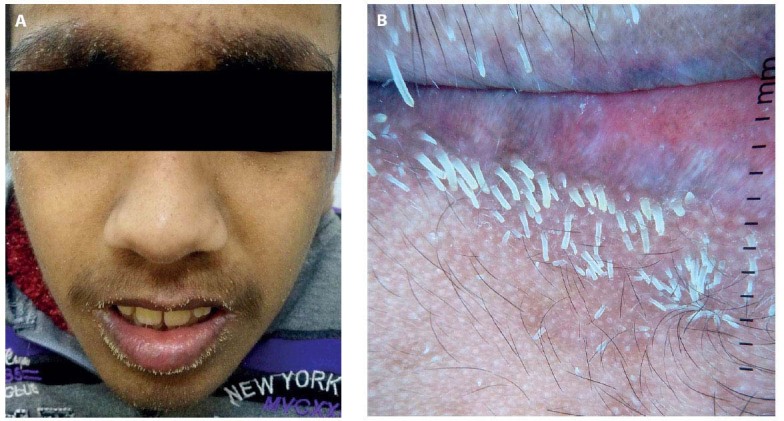
(A) Filamentous, off-white to yellowish follicular growths in the perioral region, eyelashes, eyebrows, nose, and cheeks. (B) Uniform, cylindrical, solid, off-white colored deposits encircling normal hair follicles seen on non-polarized dermoscopy.

A 16-year-old boy with a known case of autism spectrum disorder presented with complaints of multiple yellowish white deposits over hair-bearing areas of his face for the previous 3 years. On examination, there were multiple filamentous, off-white to yellowish, follicular growths in his perioral region, eyelashes, eyebrows, nose, and cheeks (Figure 1A). Dermoscopy revealed uniform cylindrical, solid, off-white colored deposits encircling normal hair follicles (Figure 1B). The gram stain and potassium hydroxide preparations were negative. The growths were clinically diagnosed to be sebaceous filaments.

## Teaching Point

Sebaceous filaments are yellowish to off-white collections of sebum and dead cells around hair follicles. These are usually found in normal healthy individuals predominantly on the nose, and they mimic comedones and trichostasis spinulosa [1]. The tail of the *Demodex* mite is an important differential on dermoscopy. In our patient, neglect of personal hygiene and possible genetic predisposition resulted in considerable growth of these sebaceous filaments.

## References

[1] Plewig G, Wolff HH (1976). Arch Dermatol Res.

